# Examining the role of a decision aid in reducing decisional conflict amongst hospital healthcare workers towards receiving the influenza vaccine

**DOI:** 10.1186/s12913-016-1339-0

**Published:** 2016-03-09

**Authors:** Holly Seale, Rajneesh Kaur, Kerryn Lajoie, Julie Dixon, Julie Gallard

**Affiliations:** School of Public Health and Community Medicine, UNSW Medicine, University of New South Wales, Sydney, Australia; Immunisation Services, Melbourne, Victoria Australia; Planning and Population Health, South Eastern Sydney Local Health District, Sydney, Australia; Centre for Hospital Epidemiology and Staff Services, Prince of Wales Hospital, Sydney, Australia

**Keywords:** Decision aid, Influenza vaccination, Healthcare workers, Hospital, Qualitative

## Abstract

**Background:**

Currently the uptake of the influenza vaccine amongst Australian hospital staff remains low. While some staff members choose not to receive the vaccine, others may feel decisional conflict around whether to receive the vaccine or not. Having access to information that is personalized to the staff members’ concerns may alleviate this conflict. Our study aimed to explore the attitudes of hospital staff towards an online decision aid (DA), which focuses on influenza and the vaccine. We were also interested to examine whether they accepted the new tool and whether they had any suggestions for improvements.

**Methods:**

Forty-one semi-structured interviews were undertaken with a range of hospital staff from two major public hospitals in Sydney and Melbourne, Australia in 2013. Emails and posters were used to inform staff members about the study. Thematic analysis was performed to explore the attitudes of hospital staff towards the DA.

**Results:**

Our participants were well aware of the time/location of the staff vaccination clinics, however very few reported attending or receiving any educational material about the disease or the vaccine. Amongst those who did receive material, they felt that the messages were “dumbed down”. There was a mostly positive response to the DA from participants, however they felt that unless it was included as part of mandatory training or orientation, it would be difficult to get staff to use the tool.

**Conclusions:**

Previous studies have established that education is an important component of an influenza vaccination program. We believe that the decision aid offers an alternative approach to delivering balanced information to staff members, which may reduce workload burdens on administrators and drive up rates.

## Background

Previous studies have suggested that healthcare workers (HCWs) experience decisional conflict or uncertainty of the best alternative when deciding about influenza immunization [1–3]. Decisional conflict arises from two sources. First, people are uncertain because of the inherent difficulty of the choice they face, where there are potential advantages but also potential disadvantages. The second source includes modifiable factors that make an inherently difficult decision even more difficult. These include: lack of knowledge, unclear values, social pressure, and a lack of support or skills or other resources [4, 5]. As a result, the individual has difficulty deciding which option to choose and it can lead to an inability to perform effective decision-making. Such conflict often occurs with many health-related decisions since options affecting ones health often have desirable outcomes but also potential risks as well. Decisional conflict, however, can occur even with health decisions where there is clear evidence that the benefits will outweigh the risks. In regards to influenza vaccination, this conflict may be associated with misperceptions about influenza (i.e. perceived personal risk, transmission, and severity) or about the vaccine (i.e. safety, effectiveness, and necessity).

To help alleviate this decisional conflict experienced by HCWs, healthcare organizations must incorporate an education component into their annual influenza vaccination campaigns. We propose that hospitals and other healthcare organizations consider the use of a decision aid (DA) for their staff. A DA is a tool which has been used in a variety of health settings to help an individual understand their options and to assess the risks and benefits of a particular behavior or treatment. This allows them to consider the options from a personal view (e.g. how important the possible benefits and harms are to them) and it prepares them to participate in making a decision. A DA may be in the form of a pamphlet, video, or web-based tool. Researchers have shown that this non-directive educational approach can help individuals to reach a decision by alleviating their ambivalence and in doing so, elicit the desired behavior change [6–9].

We theorized that a DA for influenza vaccination will improve the quality of decision making including: increasing the HCWs knowledge about the pathogen and the vaccine and their comprehension about the options available to protect them against influenza including vaccination; help with their expectations about the potential outcomes with and without vaccination and reduce their decisional conflict regarding choice. Currently, there is limited evidence about the use of DA’s for non-patient groups and even less literature around the level of acceptability of a DA that is targeted at HCWs. Therefore this study aimed to explore the attitudes of hospital staff members towards the current approaches used to educate and promote the influenza vaccine and to examine their feelings and level of acceptability towards an online influenza vaccination DA.

## Methods

### Study design

Forty one semi-structured interviews were undertaken with hospital staff from two major public hospitals in Sydney and Melbourne, Australia in 2013. The study was approved by the Human Research Ethics Committee (HREC) of the South Eastern Sydney Local Health Distract-Northern Sector (SESLHD-NS) and the Human Research Ethics Committee, Melbourne Health.

### Participants

Posters, all-user/ward specific emails and snowballing were used to notify staff at the two sites. Participation in the study was open to a relatively broad demographic and we continued to recruit staff members who volunteered for the study until we reached the point of saturation (clinical and non-clinical staff). While we did not set recruitment quotas by staff category, we did purposefully reach out to some ward directors (Emergency department, surgical wards) and asked them to alert their clinical staff members about the study. At the time of recruitment, we were unaware of the staff member’s influenza vaccination history. Participants received a pre-paid giftcard (AU$50) as compensation for their involvement. Participants were only included into the study when full written consent had been received. As part of the consent process, participants were provided with a participant information sheet, which outlined the purpose and nature of the study. The interviews were conducted on site at the hospitals.

### Decision aid development

The online DA was hosted on the University of New South Wales server and was accessed via the dedicated web link. The DA was designed by HS and developed based on the patient DA resources available from the Ottawa Hospital Research Institute website (http://decisionaid.ohri.ca/index.html) and from the International Patient Decision Aid Standards (IPDAS) Collaboration (http://ipdas.ohri.ca/). The content was informed by a review of the published literature, and was aligned with the information provided in The Australian Immunisation Handbook [10]. Prior to use, the DA was reviewed by two practicing doctors and two public health immunization experts. Updates were made based on their feedback.

### Utilizing the decision aid

From the home screen, participants had the option of clicking three links which directed them to either: (1) information about influenza (general facts about influenza i.e. transmission, contagiousness, and complications); and about the epidemiology of influenza in Australia; (2) the influenza DA; or (3) information about the study. Participants were unable to commence the DA until they had indicated whether they wanted to receive the vaccine or not. Once started, they navigated through five screens of information which was presented with the use of drop down tabs, dot points and colored boxes (Table 1). The next two pages included 11 questions which presented staff with reasons for/against receiving the vaccine and asked them to nominate whether they felt it was important to them. Participants were unable to move forward in the DA unless they answered the questions. Finally the last page presented staff with an opportunity to indicate whether they had decided to receive the vaccine or not. Once complete, a summary page appeared which listed the responses entered into the DA by the participant. The DA also allowed the staff member to enter an email address if they wished to be followed up separately by staff from the staff health clinic.

**Table 1:** Information presented in the online decision aid

Section	Topics covered
General information about the vaccine	How the vaccine works; seasonality/matching; effectiveness; development of antibodies; factors impacting on level of protection; vaccine composition, adverse effects
Impact of hospital staff vaccination	1. Evidence supporting the use of the influenza vaccine in hospitals^a^
	2. Rational for vaccinating HCWs including information about asymptomatic transmission, absenteeism’s and impact of influenza on staff performance^a^
Risks associated with influenza and with the influenza vaccine	Clinical features [10]
	Complications of influenza [10]
	Risks associated with the vaccine [10]
	Contraindications [10]
Advantages of getting vaccinated	Protection from acquiring the infection from patients/colleagues
	Protection for other staff members, patients, family/friends
	Reducing absenteeism
	Reducing the risk of taking influenza home
	Protection during outbreaks
Disadvantages of getting vaccinated	Potential for fever, malaise and myalgia
	Potential for local and immediate adverse events
	Inconvenience of getting vaccinated

^a^Published literature referenced

### Data collection

An interview guide was developed by the lead author and reviewed by the researchers to identify key areas of interest for the study. The guide was used to start the discussions with participants and was not followed absolutely. The interviews focused on general attitudes and perceptions towards: the influenza vaccine; and the strategies used by the hospital to educate and promote the vaccine. We explored the participants information and decision making needs. We also examined the participant’s feelings and level of acceptance towards the usefulness, and the content and style of the online DA we had developed. Interviews were conducted after the participant had reviewed the online DA. During the interview, paraphrasing and additional questions were added to seek clarification. This was to ensure that the study included most of the opinions and was flexible to changes depending on the actual scenario. During the interviews, member checking was conducted to ensure that the codes identified during the early phase of analysis were appropriate. The interviews were undertaken by two trained researchers HS (PhD, Senior Lecturer) and RK (MPH, PhD Student), audio-recorded and the first 24 professionally transcribed verbatim. The remaining interviews were closely reviewed for the emergence of additional themes but were not transcribed. Repeat interviews were not conducted

### Data analysis

The interviews were analyzed thematically. Two investigators developed a list of themes after one quarter of the transcripts had been analyzed. An agreed framework was then applied to another subsample of transcripts and modified further. Using this final framework, all of the transcripts were analyzed and coded. Text was organized within the identified themes of the developed framework without the use of any software.

## Results

A total of 41 interviews were undertaken between March and May 2013 (Sydney: 24, Melbourne: 17). The demographic characteristics of the study participants are summarized in Table 2. The interview results are presented below according to the themes that were identified.

**Table 2:** Demographic characteristics of the forty-one participants

Characteristics	N(%)
Gender	
Male	13 (31.7)
Female	28 (68.3)
Age group	
< 24 years	2 (4.8)
25–34 years	17 (41.5)
35–44 years	9 (22)
45–54 years	8 (19.5)
55–64 years	4 (9.8)
65 years	1 (2.4)
Number of years of working in healthcare (years)	
Median (range)	12 (1–43)
Number of years of working in current position (years)	
Median (range)	3(1–29)
Primary employment status	
Resident/Registrar/Provisional Fellow	10 (24.4)
Staff Specialist – (>/0.5 FTE)	3 (7.3)
NUM	1 (2.4)
Registered nurse	13 (31.7)
Other (field/position specified below)^a^	14 (34.2)
Uptake of influenza vaccination	
Previous receipt of the influenza vaccine	31 (75.6)
Vaccinated against influenza in:	
2013	7 N
2012	6 (14.6)

*Abbreviations*: *NUM* nurse unit manager, *FTE* full time equivalent

^a^(Intern, Clinical Nurse Coordinator, Enrolled Nurse, Allied health, Administrator, Finance, Clinical Assistant, Medical Scientist, Phlebotomist)

### It’s important to get the flu vaccine, but I don’t need it!

Before asking participants questions regarding the use of the DA, we wanted to have an understanding of their general attitudes towards the vaccine. While there were high levels of support amongst participants regarding the general use of the influenza vaccine, not everyone felt a personal need to get vaccinated. Interestingly, there was confusion amongst some participants who referred to the influenza vaccine as being ‘new’ and believed that uptake levels were low because: *“there hasn’t been as much society acceptance of it”* (Registered Nurse).

*I haven’t got the vaccine before. I guess I’ve got concerns about … one is the side effects of the vaccine and two is about how effective it is and, you know, whether it covers all strains of potential viral respiratory illnesses* (Resident/Registrar).

### Not paying attention to the signs

Participants were asked to reflect on their exposures to hospital health promotion messages around the influenza vaccine. Participants highlighted that they were well informed about clinic opening/closing times; when the mass clinics were being run etc. but spoke very little about seeing any other messages about the vaccine/disease.

*I think people don’t pay attention to signs. There’s too many of them* (Resident/Registrar).

*The main purpose of the campaign that I can tell is just to tell the dates. People I know who get flu vaccine, they want to get it, and we all go and get it together. I don’t even look at the posters. I just see the… flu vaccine, dates, that’s it I’m done* (Resident/Registrar).

Amongst the staff members interviewed, there was a sense that they had not received or reviewed any educational material and amongst those who did receive material, they felt that the messages were “dumbed down”. One participant even went on as far as to say that the people responsible for the campaigns “*should treat healthy people with a bit more respect and give them more information*” (Allied Health).

Lastly, attendance at the hospital delivered education sessions (either delivered on the ward or at grand rounds) about the vaccine was stated to be low, with participants highlighting that the last time they had received any information about the vaccine was during their university training. While others said that they sought information about the vaccine from the internet.

*I picked up on the posters because that’s what I’ve noticed recently. They evidently do hold one’s eye as you’re passing them, but whether or not they give the momentum to somebody to then go forward and look into it a little bit more, as I hope I’ve done now, they perhaps don’t give enough reason on the posters I don’t think to go ahead and say, “Well, perhaps I should do the right thing and go and have that done this year* (Clinical Nurse Consultant).

### I didn’t know or realize that

Prior to viewing the DA, participants were asked to nominate topics that they felt they needed information on. Reported gaps in their knowledge about the virus included: how it transmits and how long a person is infectious for; the epidemiology of influenza in Australia and the risk of influenza for HCWs. Information gaps about the vaccine included: how the vaccine is developed and how the strains are selected, the effectiveness of the vaccine, the time required to build immunity post immunization, and about the safety of the vaccine/side effect profile. Other participants were uncertain about the nature of the vaccine (e.g. whether it was live or attenuated) and about how many strains are included. Participants were surprised to read about the potential for asymptomatic carriage, about the different age groups at risk (i.e. the young vs. very old), about the link between influenza and hospital absenteeism.

*The other thing I didn’t know was that it affected children. I thought it was mostly for immunocompromised people or elderly…I was surprised to see those statistics that children are most at risk* (Registered Nurse).

*I thought it only came in one strain and not that every year it’s modified to what, like, the strain they expect for that year. I thought that people always got sick after they had the injection and not that the vaccine takes up to two weeks for it to become effective.* (Registered Nurse).

After viewing the DA, participants felt they still wanted more information about: (1) the epidemiology of the influenza in Australia, (2) the risk of influenza for certain wards/occupations (i.e. staff in emergency vs. the surgical department) and (3) the risks/benefits of vaccinating pregnant women.

### Acceptability of the DA

There was a mostly positive response to the DA from participants. They spoke about “feeling more informed”, “feeling reoriented to the issues” and “feeling that they learnt something new from the DA”. One participant summarized the impact by saying that *“the poster is telling you to go and have the needle, the DA is giving you information why you should have the needle”* (Student Nurse). Some participants even went as far as to say that it made them inspired to get vaccinated. However, this feeling was not unanimous as other participants remarked that the DA did not change their decision (to decline vaccination) but it did however make them feel that their decision was more informed.

*I think it’s a nice way of reorienting me as a health professional… It wasn’t rubbing my nose in it…. I’m glad I’ve read a little bit more today. That’s been more of a reminder. I’ve not been to any education sessions until back in the day. I’ve been trained an awful long time and not had any for many years* (Clinical Nurse Consultant).

*I felt pretty confident with my knowledge about influenza, but I did learn something from the DA and that was that some people can be asymptomatic and have it and still shed the virus and infect the patients; which, thinking about it, I understand that now* (Resident/Registrar).

The frequently asked questions (about the disease and the vaccine), the pros/cons of getting vaccinated vs. not getting vaccinated, and the short summaries of the findings from the research trials around influenza vaccine effectiveness were some of the positive aspects of the DA nominated by participants. Having information presented in dot points, click down screens and pop-up boxes were nominated as being useful by the participants. Only one participant felt that the information was not balanced and that it was “biased towards making people go and get the vaccination”.

### It needs to be compulsory

Making the DA accessible via the hospitals intranet was suggested as one way of providing access to the staff. Alternative approaches included emailing out the DA to all staff members or making it available on the desktop of ward computers. However, the issue of staff not having access to a computer in the workplace was suggested as an obstacle, as was the issue of having sufficient time to go through the DA.

The majority of participants were in favor of making the DA a compulsory part of orientation for new staff members. One participant suggested that staff members should be able to obtain continuing professional development (CPD) points by reviewing the DA, while another participant suggested that completing the DA should be thought of as no different to undertaking compulsory fire training.

*I think your hurdles are probably getting people to use it, just time wise. I think if it was compulsory … a compulsory staff wide survey that would be the best way to just get it out there* (Registered Nurse).

*I don’t think asking staff mandatory things are unreasonable. We do mandatory fire training, we do mandatory aggression management, and we do all these things that we have to complete* (Registered Nurse).

However, someone else questioned the legitimacy of making the DA a mandatory requirement when the vaccine is not: “*If you’re not going to mandate the vaccination but you’re going to mandate the reading of that that sounds like coercion. You know, so if that, if that exists then why not just make people get the vaccination in the first place?”* (Registered Nurse).

## Discussion

Using qualitative methods, this study explored the opinions of hospital staff towards the DA and their acceptability of the new tool. Amongst our participants, there was a positive response towards the DA and its use as an educational tool in the hospital. To the best of our knowledge, we are the second group worldwide to explore the use of a DA for hospital HCWs and the first in Australia. In 2007, McCarthy et.al was the first group to develop a DA and evaluate it with a small group of HCWs in in Ontario, Canada [11]. They found that their participants believed that the tool helped them recognize that a decision needed to be made and that it was dependent on what matters most to them. During the 2009/10 influenza season, the researchers went onto explore the impact of the DA on HCWs working in a multi-service, non-acute care healthcare organization [12]. They reported a statistically significant (*P* = 0.020) greater improvement in confidence in immunization decision in the arm randomized to the DA compared to the arm randomized to control. They also observed a statistically significant decrease in individuals being unsure about their immunization decision in the arm that received the DA [12]. While we did not set out to test the impact of the DA on intention to immunize, some participants did declare that they felt “inspired” to get vaccinated after reviewing the DA. However given the purposive sampling approached use to recruit participants we cannot rule out the potential for confounding effects on these findings.

It is now well established that influenza vaccination campaigns based solely on voluntary or educational interventions will not increase coverage in the hospital setting beyond 70 to 80 % [13]. However, voluntary campaigns that incorporate education simultaneously with other interventions (i.e. notification and free provision of vaccination) do yield results [1]. As advocated by Hollmeyer et al. a successful program must include culturally sensitive education on the risk of influenza and the overall benefits of vaccination, tailored to specific professional characteristics [14]. From our interviews, there was a sense that staff members were not receiving tailored information about influenza or about vaccine or about the rational for vaccination. Amongst those who had received educational materials, the response was not positive, with some staff members describing the material as being “dumbed down”. Amongst the staff members that we spoke to there was an expressed need for further information about the epidemiology of influenza, about risks associated with the infection, about how the strains are selected for the vaccine and how it is developed. For HCWs who have an underlying illness or who are pregnant, they may need to receive additional information and reassurances about the safety of the vaccine from someone who knows their health situation. Hospitals don’t currently cater well for these individuals and it is important that tools such as the DA include information for these subgroups [15].

The issue for many hospitals is how to successfully deliver education campaigns to hospital staff, which include medical and non-medical employees (including all terms of employment e.g. full-time, part-time, volunteers, contractors, students). Promotional emails (mass vs. personalized), plenary seminars, peer-to-peer education sessions, newsletters/bulletins and ward based consultations with occupational health staff are just some of the approaches we have observe being used to educate staff members about the need to get the influenza vaccine [16]. Based on our experience and the responses from our participants, we theorize that there are a number of issues associated with these type of approaches including: (1) they require large amounts of staff time and resources; (2) they may not have the reach to capture all categories of staff, from all disciplines and employment types (especially those who work off campus); (3) they may not have sufficient content to satisfy the information needs of staff, or they may be inappropriately pitched; and (4) staff responsible for the delivery may not be appropriately trained to execute effective communication campaigns [16, 17].

There are many advantages associated with online learning approaches to education. Firstly, participation can be easily monitored and documented, unlike attendance at in-services or via the passive delivery of educational materials. The system can be hosted on the hospitals intranet or home page and links can be provided via on-ward computers and emails. In addition, the ability to access the DA from any Internet location also allows for the participation of providers with varying demands on their time and differing work locations (especially relevant for those who work off-site). Through the use of drop down screens, dot points, figures and links to further information, the DA can provide information in such a way that it is appropriate for staff with and without medical training. In addition, it can be personalized to staff members with specific health conditions i.e. pregnant women, older staff members or those with underlying conditions. It provides an option for staff members interested in finding out more about the infection or vaccine to access additional readings and other resources. Lastly, capturing the responses to the options given in the DA via an electronic database allows for analysis of factors associated with uptake and refusal of the vaccine. This information could be integrated into future institutional campaigns.

The use of in-depth interviews to elicit a greater depth in the information is the key strength of this study. However, interviews were only undertaken with a self-selecting group of participants, so the possibility of other important themes emerging cannot be ruled out. The possibility of response bias cannot be eliminated, as HCWs who held a more negative attitude about the influenza vaccination may have been more willing to participate. We were unable to compare the characteristics of the participants with those of the general hospital population. This was a small, qualitative study, therefore confirmation of the findings via a survey tool is recommended among a large representative population of HCWs to get further insight into whether a DA would benefit.

## Conclusions

The aim of this initial study was to explore the attitudes of hospital staff towards the DA and their acceptability of the new tool. We were particularly interested to establish whether the pitch, length, style and content of the DA were attractive and appropriate to staff members. Based on the interviews, our participants were receptive to the DA and only had minor suggestions for improvements. Following these improvements, we plan to undertake a randomized controlled trial to ascertain whether its use leads to changes in the level of confidence amongst HCWs and their intentions to get vaccinated and ultimately changes in vaccine uptake in the hospital setting.
